# Additional gastrectomy in early-stage gastric cancer after non-curative endoscopic resection: a meta-analysis

**DOI:** 10.1093/gastro/goz007

**Published:** 2019-03-08

**Authors:** Run-Cong Nie, Shu-Qiang Yuan, Yuan-Fang Li, Shi Chen, Yong-Ming Chen, Xiao-Jiang Chen, Guo-Ming Chen, Zhi-Wei Zhou, Ying-Bo Chen

**Affiliations:** 1 Department of Gastric Surgery, Sun Yat-sen University Cancer Center, Guangzhou, Guangdong, P. R. China; 2 State Key Laboratory of Oncology in South China, Guangzhou, Guangdong, P. R. China; 3 Collaborative Innovation Center for Cancer Medicine, Guangzhou, Guangdong, P. R. China; 4 Department of Gastric Surgery, The 6th Affiliated Hospital of Sun Yat-sen University, Guangzhou, Guangdong, P. R. China

**Keywords:** Early gastric cancer, non-curative, endoscopic resection, gastrectomy

## Abstract

**Background and objective:**

The role of additional gastrectomy after non-curative endoscopic resection remains uncertain. The present meta-analysis aimed to explore the risk factors for early-stage gastric-cancer patients after non-curative endoscopic resection and evaluate the efficacy of additional gastrectomy.

**Methods:**

Relevant studies that reported additional gastrectomy after non-curative endoscopic resection were comprehensively searched in MedLine, Web of Science and EMBASE. We first investigated the risk factors for residual tumor and lymph-node metastasis after non-curative endoscopic resection and then analysed the survival outcome, including 5-year overall survival (OS) and 5-year disease-free survival, of additional gastrectomy.

**Results:**

Twenty-one studies comprising 4870 cases were included in the present study. We found that residual tumor was associated with larger tumor size (>3 cm) (odds ratio [OR] = 2.81, *P* < 0.001), undifferentiated tumor type (OR = 1.78, *P* = 0.011) and positive horizontal margin (OR = 9.78, *P* < 0.001). Lymph-node metastasis was associated with larger tumor size (>3 cm) (OR = 1.73, *P* < 0.001), elevated tumor type (OR = 1.60, *P* = 0.035), deeper tumor invasion (>SM1) (OR = 2.68, *P* < 0.001), lymphatic invasion (OR = 4.65, *P* < 0.001) and positive vertical margin (OR = 2.30, *P* < 0.001). Patients who underwent additional gastrectomy had longer 5-year OS (hazard ratio [HR] = 0.34, *P* < 0.001), 5-year disease-free survival (HR = 0.52, *P* = 0.001) and 5-year disease-specific survival (HR = 0.50, *P* < 0.001) than those who did not. Moreover, elderly patients also benefited from additional gastrectomy regarding 5-year OS (HR = 0.41, *P* = 0.001).

**Conclusions:**

Additional gastrectomy with lymph-node dissection might improve the survival of early-stage gastric-cancer patients after non-curative endoscopic resection. However, risk stratification should be performed to avoid excessive treatment.

## Introduction

Although the incidence of gastric cancer is decreasing, it remains the fourth most common malignancy worldwide, with an estimated 951,600 new cases in 2012 [1]. With a widespread increase in medical checkups and endoscopic screenings, the proportion of early-stage gastric cancer (EGC) is increasing, particularly in Japan [2] and Korea [3].

EGC is defined as gastric cancer with lesions confined to the mucosa or submucosa, irrespective of the status of lymph-node metastasis [4]. The treatment of EGC is generally minimally invasive, with the increasing use of endoscopic resection, which comprises endoscopic mucosal resection (EMR) and endoscopic submucosal dissection (ESD). EMR and ESD have been shown to be effective treatments for EGC patients without lymph-node metastasis [5, 6]. However, with the widespread use of endoscopic resection, the number of patients receiving endoscopic resection that does not meet the curative criteria described by the Japanese Gastric Cancer Treatment Guidelines (i.e. non-curative) [7] is increasing. For non-curative endoscopic resection, additional gastrectomy with lymph-node (LN) dissection is recommended to remove the residual tumor and suspicious lymph nodes. However, the rates of residual tumor and lymph-node metastasis (LNM) after additional surgery have been reported to be low (5.0%–13.4% for residual tumor [8–11] and 4.3%–12.7% for LNM [8–20]). Moreover, some patients refuse additional gastrectomy after non-curative endoscopic resection due to old age and concomitant diseases, which may lead to shorter overall survival (OS). Suzuki *et al.* [16] reported that patients who underwent additional gastrectomy with LN dissection had longer 5-year OS than patients who underwent only follow-up (94.75 vs. 83.8%, *P* < 0.001), whereas the 5-year disease-specific survival (DSS) was not significantly different between the two groups (98.8% vs. 96.8%, *P* = 0.100). However, Hatta *et al.* [18] demonstrated a large difference regarding OS and DSS between the two groups. Previous studies on patient survival following additional gastrectomy have reported inconsistent results [9, 13, 14, 16, 18, 21–25].

Therefore, this meta-analysis was performed to review the additional gastrectomy for non-curative endoscopic resection, to investigate the risk factors for residual tumor and LNM after non-curative endoscopic resection, and to explore whether EGC patients benefit from additional gastrectomy regarding survival outcome.

## Methods

### Search strategy

On 4 October 2017, the studies on gastric cancer and endoscopic resection with restrictions in English language were systematically searched in MedLine, Web of Science and EMBASE databases. The following terms in [Title/Abstract] were used: “endoscopic resection”, “endoscopic submucosal dissection”, “endoscopic mucosal resection”, “early gastric cancer”, “early gastric carcinoma”, “early gastric neoplasm”, “early stomach cancer”, “early stomach neoplasm”, “gastrectomy”, “surgery” and “surgical procedure”. We also screened the reference lists of the retrieved studies manually to supplement the related studies.

### Inclusion and exclusion criteria

According to the Japanese Gastric Cancer Association (JGCA) [7], the endoscopic resection is considered curative if the resection conforms to the following criteria: en bloc resection, tumor size ≤2 cm, differentiated type, pT1a, negative horizontal and vertical margins, no lymphatic invasion and no venous invasion. Non-curative endoscopic resection was defined as resection that does not meet the above criteria.

All relevant studies that compared additional gastrectomy with non-gastrectomy after non-curative endoscopic resection were included. Studies that explored the risk factors of residual tumor and/or LNM after additional gastrectomy were also included. Animal studies, case reports, letters, comments, meeting abstracts, reviews and clinical studies with fewer than 15 patients were excluded. If multiple articles reported the same patient cohort, the articles containing more recent and complete data were selected. Multiple articles were included if they reported different endpoints of the same patient cohort.

### Data extraction

Two authors (R.C.N. and Y.Q.Y.) extracted the following information from each study independently: author, publication year, country, study period, study design, patient number and median follow-up period. We also collected the clinicopathological characteristics of the two groups. Survival outcomes of these studies included 5-year OS, disease-free survival (DFS) and DSS. If the studies did not report the 5-year survival rate, we calculated the hazard ratio (HR) from the survival curves, as described by Parmar *et al.* [26]. The two senior authors (Y.F.L. and Y.B.C.) resolved any disagreements in the extracting data.

### Quality assessment

All studies in this meta-analysis were retrospective; thus, the quality of the included studies was assessed with the Newcastle–Ottawa Scale (NOS) [27] using a scale from 0 to 9. Studies with a score higher than 5 were considered high-quality studies in this analysis.

### Statistical analysis

All the data in this meta-analysis were analysed using STATA/SE 12.0 (StataCorp LP, College Station, TX, USA). The weighted mean difference and odds ratio (OR) with reported 95% confidence intervals (CIs) were used to analyse continuous and dichotomous data, respectively. HRs were used to assess the treatment effect of additional gastrectomy compared with non-gastrectomy. In the present study, we investigated the related risk factors with tumor residual and LNM for the patients in the additional gastrectomy group. All statistical analyses were performed with the χ^2^ test using the *I*^2^ statistic to assess heterogeneity, with the level of significance set at 10%. The fixed-effects model was used when there was no significant heterogeneity. Otherwise, the random-effects model was used. Publication bias was tested using funnel plots.

## Results

### Characteristics of the included studies

**Figure 1.**
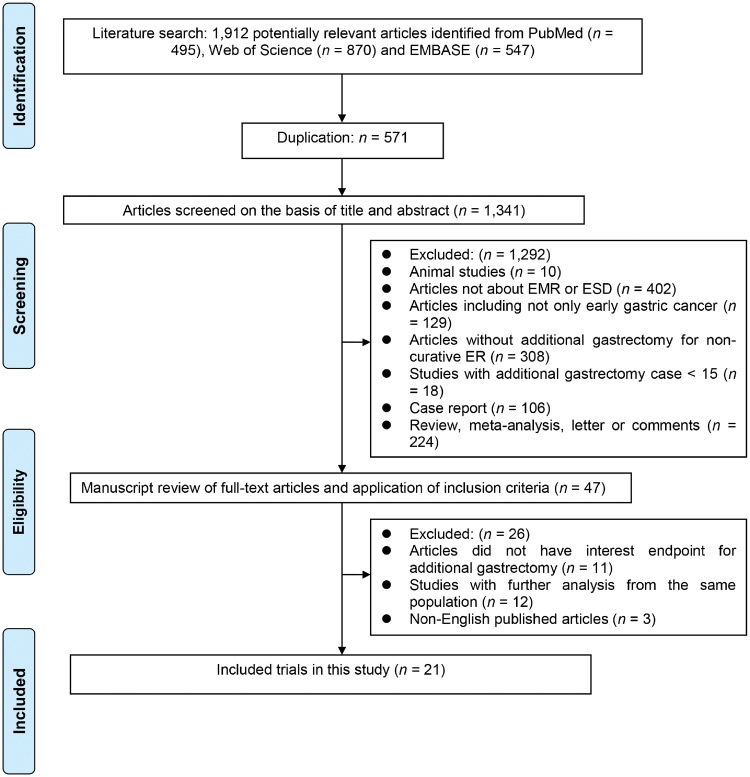
Study flow diagram of the included studies. EMR, endoscopic mucosal resection; ESD, endoscopic submucosal dissection; ER, endoscopic resection.

After the systematic search, a total of 21 studies [8–20, 22, 24, 25, 28–32] comprising 4,870 cases were included in the final analysis (Figure 1). The characteristics of the included studies are shown in Table 1. All the included studies were retrospective and primarily considered Japanese and Korean patient populations. Eleven studies [9, 13, 14, 16, 18, 22, 24, 25, 29, 31, 32] performed survival data collection. These studies explored the survival differences between gastrectomy and observation but did not compare gastrectomy with other treatment strategies (such as adjuvant treatment and re-ESD). Five studies [8, 11, 19, 28, 30] reported the risk factors for residual tumor after additional gastrectomy and 11 studies [8–12, 14–17, 19, 20] reported the risk factors for LNM. All included studies were considered high-quality.

**Table 1.** Characteristics of the included studies

Author, year	Country	Study period	Study design	Patients (G/NG)	Median follow-up (months)	Quality scores	Endpoints
Lee, 2010 [28]	Korea	2006–2009	R	28/–	NA	5	1
Kusano, 2011^a^ [29]	Japan	1999–2005	R	38/82	G: 43.2	5	3, 4
					NG: 38.1		
Son, 2013 [15]	Korea	2001–2011	R	147/–	NA	5	2
Ito, 2013 [8]	Japan	2001–2012	R	41/–	NA	5	1, 2
Park, 2013 [12]	Korea	2003–2012	R	102/–	NA	5	2
Choi, 2015 [13]	Korea	2003–2010	R	28/61	NA	5	3, 5
Kim, 2015^b^ [9]	Korea	2000–2011	R	194/80	60.5	7	2, 3
Noh, 2015 [22]	Korea	2005–2013	R	45/38	NA	6	5
Yang, 2015 [14]	Korea	2005–2013	R	123/144	40.7	7	2, 4
Suzuki, 2016^a^ [16]	Japan	1999–2010	R	356/212	74.0	7	2, 3, 4
Ishii, 2016 [17]	Japan	1997–2013	R	112/–	NA	5	2
Toya, 2016 [24]	Japan	2002–2010	R	45/21	G: 93.6	7	3, 4
					NG: 70.8		
Hatta, 2016^c^ [18]	Japan	2000–2011	R	1064/905	G: 67.0	7	3, 4
					NG: 64.0		
Hatta, 2017^c^ [20]	Japan	2000–2011	R	1101/905	G: 67.0	7	2
					NG: 64.0		
Hwang, 2017 [30]	Korea	2003–2013	R	80/–	NA	5	1
Sumiyoshi, 2017 [25]	Japan	2003–2010	R	15/17	G: 73.0	7	3
					NG: 62.0		
Sunagawa, 2017 [11]	Japan	2005–2015	R	200/–	25.3	5	1, 2
Kim, 2017 [19]	Korea	2004–2014	R	350/–	NA	5	1, 2
Jung, 2017^d^ [10]	Korea	2007–2015	R	321/–	NA	5	2
Jeon, 2017^d^ [31]	Korea	2007–2016	R	264/198	G: 84.8	7	4, 5
					NG: 70.8		
Pyo, 2017^b^ [32]	Korea	2000–2013	R	87/51	G: 37	5	3, 4, 5
					NG: 30		
							

R, retrospective study; G, additional gastrectomy group; NG, non-gastrectomy group; NA, not available; 1, residual tumor; 2, lymph-node metastasis; 3, overall survival; 4, disease-free survival; 5, disease-specific survival.

^a^Kusano *et al.* reported the elderly patient cohort of the study of Suzuki *et al.* Therefore, the former was only included for subgroup analysis of the elderly patients, and the latter was included for the entire cohort.

^b^Kim *et al.* explored the role of additional surgery for non-curative endoscopic resection of EGC, whereas Pyo *et al.* later reported long-term outcomes after non-curative endoscopic resection in elderly patients with EGC at the same institution. In the present study, the former was included for the total population, whereas the latter was included for subgroup analysis of the elderly patients.

^c^Hatta *et al.* reported different endpoints in 2016 and 2017.

^d^The studies reported by Jung *et al.* and Jeon *et al.* were from the same patient cohort with different endpoints.

### Risk factors for residual tumor

As shown in Table 2, patients with larger tumor size (>3 cm) (OR = 2.81, 95% CI = 1.90–4.18, *P* < 0.001; *I*^2^^ ^= 0, *P* = 0.469), undifferentiated tumor type (OR = 1.78, 95% CI = 1.14–2.77, *P* = 0.011; *I*^2^^ ^= 0, *P* = 0.413) or positive horizontal margin (OR = 9.78, 95% CI = 6.30–15.18, *P* < 0.001; *I*^2^^ ^= 44.3%, *P* = 0.145) exhibited higher rates of residual tumor than their controls. Gross type, ulcer formation, tumor depth (SM1 stage), lymphatic invasion and vertical margin were not associated with residual tumor.

**Table 2.** Association between clinicopathological characteristics and residual tumor in the additional gastrostomy group

Variable	No. of studies	Statistic		Heterogeneity	
		OR (95% CI)	*P*-value	*I*^2^	*P*-value
Tumor size (>3 cm)	5 [8, 11, 19, 28, 30]	2.81 (1.90–4.18)	<0.001	0	0.469
Gross type (elevated type)	3 [8, 11, 30]	0.55 (0.29–1.06)	0.076	0	0.936
Ulcer formation	3 [8, 11, 19]	0.84 (0.36–1.92)	0.671	0	0.849
Tumor depth (SM1 stage)	4 [8, 11, 19, 30]	1.05 (0.13–8.30)	0.966	94.0%	<0.001
Undifferentiated type	5 [8, 11, 19, 28, 30]	1.78 (1.14–2.77)	0.011	0	0.413
Lymphatic invasion	3 [8, 11, 30]	1.95 (0.94–4.04)	0.071	0	0.369
Horizontal margin	4 [8, 11, 19, 30]	9.78 (6.30–15.18)	<0.001	44.3%	0.145
Vertical margin	3 [8, 11, 19]	2.61 (0.85–8.03)	0.094	72.6%	0.026

OR, odds ratio; CI, confidence interval; SM1 stage, tumor invasion <500 µm into the submucosa.

### Risk factors for LNM

As shown in Table 3, LNM was found to be significantly associated with larger tumor size (>3 cm) (OR = 1.73, 95% CI = 1.30–2.32, *P* < 0.001; *I*^2^^ ^= 0, *P* = 0.493), elevated gross type (OR = 1.60, 95% CI = 1.03–2.49, *P* = 0.035; *I*^2^^ ^= 16.7%, *P* = 0.306), deeper tumor invasion (>SM1 stage, defined as tumor invasion into the submucosa <500 µm from the muscularis mucosae) (OR = 2.68, 95% CI = 1.96–3.66, *P* < 0.001; *I*^2^^ ^= 14.2%, *P* = 0.312), lymphatic invasion (OR = 4.65, 95% CI = 3.16–6.84, *P* < 0.001; *I*^2^^ ^= 24.8%, *P* = 0.256), positive horizontal invasion (OR = 0.39, 95% CI = 0.22–0.69, *P* = 0.001; *I*^2^^ ^= 31.2%, *P* = 0.190) and positive vertical margin (OR = 2.30, 95% CI = 1.70–3.11, *P* < 0.001; *I*^2^^ ^= 0, *P* = 0.687). Ulcer formation and undifferentiated type were not significantly associated with LNM.

**Table 3.** Association between clinicopathological characteristics and lymph-node involvement in the additional gastrostomy group

Variable	No. of studies	Statistic		Heterogeneity	
		OR (95% CI)	*P*-value	*I*^2^	*P*-value
Tumor size (>3 cm)	8 [8, 11, 14–17, 19, 20]	1.73 (1.30–2.32)	<0.001	0	0.493
Gross type (elevated type)	6 [8, 10, 11, 14, 16, 17]	1.60 (1.03–2.49)	0.035	16.7%	0.306
Ulcer formation	6 [11, 14, 16, 17, 19, 20]	0.90 (0.61–1.32)	0.594	38.2%	0.151
Tumor depth (>SM1 stage)	10 [8–11, 14–17, 19, 20]	2.68 (1.96–3.66)	<0.001	14.2%	0.312
Undifferentiated type	9 [8, 10, 11, 14–17, 19, 20]	0.88 (0.49–1.58)	0.673	58.1%	0.014
Lymphatic invasion	5 [8, 11, 14, 17, 20]	4.65 (3.16–6.84)	<0.001	24.8%	0.256
Horizontal margin	7 [8, 10, 11, 14, 16, 17, 19]	0.39 (0.22–0.69)	0.001	31.2%	0.190
Vertical margin	8 [8, 10, 11, 14, 16, 17, 19, 20]	2.30 (1.70–3.11)	<0.001	0	0.687

OR, odds ratio; CI, confidence intervals; SM1 stage, tumor invasion <500 µm into the submucosa.

### Survival outcome

**Figure 2.**
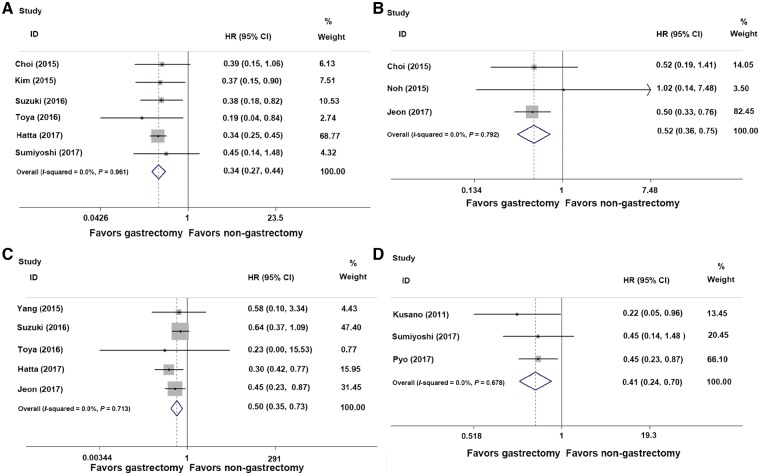
Forest plot assessing survival outcome comparing gastrectomy groups to non-gastrectomy groups. A, overall survival (HR = 0.34, *P* < 0.001); B, disease-free survival (HR = 0.52, *P* = 0.001); C, disease-special survival (HR = 0.50, *P* = 0.001); D, overall survival in the elderly patient subgroup (HR = 0.41, *P* = 0.001). HR, hazard ratio; CI, confidence interval.

Eleven studies [9, 13, 14, 16, 18, 22, 24, 25, 29, 31, 32] reported survival data for both gastrectomy and non-gastrectomy groups (Figure 2). Among these studies, six [9, 13, 16, 18, 24, 25] reported OS (Figure 2A), with no heterogeneity observed (*I*^2^^ ^= 0, *P* = 0.961). Therefore, the fixed-effects model was used. The pooled HR for 5-year OS was 0.34 (95% CI = 0.27–0.44, *P* < 0.001), indicating a better prognosis for the additional gastrectomy group.

Three studies [13, 22, 31] reported the DFS of both groups (Figure 2B), with no heterogeneity observed (*I*^2^^ ^= 0, *P* = 0.792). The pooled analysis showed that patients receiving additional gastrectomy had a longer 5-year DFS (HR = 0.52, 95% CI = 0.36–0.75, *P* = 0.001).

Five studies [14, 16, 18, 24, 31] compared the DSS between the two groups (Figure 2C). There was no obvious heterogeneity (*I*^2^^ ^= 0, *P* = 0.713) and the pooled data revealed that patients receiving additional gastrectomy had a longer 5-year DSS than patients receiving only follow-up (HR = 0.50, 95% CI = 0.35–0.73, *P* = 0.001).

Three studies [25, 29, 32] reported the OS data of elderly patients. There was no obvious heterogeneity (*I*^2^^ ^= 0, *P* = 0.678) and the pooled HR for 5-year OS was 0.41 (95% CI = 0.24–0.70, *P* = 0.001) (Figure 2D)

### Publication bias

The funnel plot indicated that there were no obvious publication biases in OS, DFS and DSS in the total population (Supplementary Figure 1). No obvious publication bias was observed in OS in the elderly patient population (Supplementary Figure 1).

## Discussion

In the present meta-analysis, we reviewed the additional gastrectomy for non-curative endoscopic resection and found that additional gastrectomy with LN dissection can prolong the 5-year OS, DFS and DSS of patients with EGC after non-curative endoscopic resection. In addition, elderly patients can benefit from additional gastrectomy regarding 5-year OS.

Several risk factors have been reported to be associated with residual tumor after non-curative endoscopic resection [8, 11, 19, 28, 30], including lymphovascular invasion and positive margins. Ito *et al.* [8] showed that tumor invasion and positive horizontal margins were associated with residual tumor. Sunagawa *et al.* [11] reported that positive horizontal and vertical margins were risk factors for residual tumor. Because of the small sample sizes of previous studies and the low incidence of residual tumor after non-curative endoscopic resection, the pooling results of our meta-analysis increased our ability to identify relevant risk factors. This meta-analysis showed that larger tumor size (>3 cm), undifferentiated tumor type and positive horizontal margins were predictors for residual tumor. Other factors, including gross type, ulcer, tumor invasion, lymphatic invasion and even vertical margin, were not predictors for residual tumor.

This study showed that the rate of LNM varies from 4.3% to 12.7% after additional gastrectomy with LN dissection. The pooled incidence of LNM was 8.0% (240/2989). These pooled results showed that patients with larger tumor size, elevated gross type tumors, deeper tumor invasion (>SM1 stage), lymphatic invasion and positive vertical margins had a higher risk of LNM. According to JGCA [7], the lack of ulcer formation is one of the best indications for EMR or ESD. Ulcer formation is considered a criterion for non-curative endoscopic resection. However, the present meta-analysis demonstrated that ulcer formation predicted neither residual tumor nor LNM. Therefore, if the resected specimen conforms to other curative criteria without histological evidence of residual tumor in the ulcer, then it is possible to regard endoscopic resection as curative.

The therapeutic strategy of a positive horizontal margin is debated. Sunagawa *et al.* [11] reported that patients with positive horizontal margins have a higher risk of residual tumor but no increased risk of LNM. Several investigators also reported that the rate of LNM is quite low when positive horizontal margins are the only indicator of non-curative surgery [14, 19]. This meta-analysis showed that positive horizontal margins are associated with a higher rate of residual tumor. Interestingly, a positive horizontal margin was negatively associated with LNM. Numata *et al.* [33] reported that all local recurrent tumors with positive horizontal margins were intramucosal lesions and could be resected by re-ESD. Given the low incidence of LNM in cases of positive horizontal margins, we consider that repeated ESD or strict endoscopic surveillance is feasible if the positive horizontal margin is the only indicator of non-curative surgery.

In this meta-analysis, we identified several risk factors, including elevated gross type, >SM1 stage and lymphatic invasion, which were associated with LNM, but not with residual tumor. Abe *et al.* [34] reported that a combination of ESD and laparoscopic LN dissection without gastrectomy for EGC patients with a risk of LNM can be effective, with minimally invasive treatments maintaining long-term outcomes. However, the sample in this previous study was very small (21 patients). Moreover, LN dissection without gastrectomy is a complex surgery. Therefore, it was still unclear whether LN dissection without gastrectomy could be performed in these patients.

In clinical practice, the decision to perform additional gastrectomy with LN dissection for non-curative endoscopic resection is influenced not only by the risk of residual tumor and LNM, but also by age, comorbidities, the wishes of the patients and their families, and the predicted quality of life after surgery. This meta-analysis showed that patients who underwent additional surgery were younger than those who did not (Supplementary Table 1). Moreover, patients who underwent additional surgery had a higher proportion of >SM1 stage tumors and lymphatic invasion, indicating that clinicians were also involved in the treatment strategy. Suzuki *et al.* [16] reported that patients who underwent additional surgery had a longer 5-year OS. However, 5-year DSS levels were similar between the two groups. Toya *et al.* [24] also found that patients who underwent additional surgery had a longer 5-year OS. However, of the patients who did not undergo additional surgery, none died of recurrent gastric cancer. Nevertheless, a multicenter retrospective study that included 1969 patients with non-curative ESD showed a larger discrepancy between OS and DSS in these two groups [18]. After pooling the survival outcome from 11 studies [9, 13, 14, 16, 18, 22, 24, 25, 29, 31, 32], our study revealed that patients with non-curative endoscopic resection could benefit from additional gastrectomy in terms of 5-year OS, DFS and DSS. Moreover, our study showed that elderly patients who underwent additional gastrectomy had a longer 5-year OS. However, additional gastrectomy should be performed selectively. Hatta *et al.* [20] established a simple but useful scoring system (‘eCura system’) to predict DSS in EGC patients after non-curative ESD. For patients at low risk of LNM categorized by the ‘eCura system’, observation may be an optimal choice because of their excellent survival outcome (5-year DSS rate, 99.6%).

There are several limitations of this meta-analysis. First, all included studies were retrospective and were performed in Japan and Korea, where the incidence of EGC is very high and endoscopic technology is advanced. Thus, whether the result of this meta-analysis applies to Western countries remains uncertain. Second, there are selection biases about treatment strategy choices before and after endoscopic resection. This bias will probably include the health of the patient and the surgeons’ estimation of the risk of LNM. Third, some of the considered studies investigated non-curative ESD patients only, whereas others reported the results of both non-curative ESD and EMR. Fourth, we could not compare the outcome between gastrectomy and other treatment strategies, such as re-ESD and adjuvant chemotherapy. Fifth, the median follow-up for non-curative endoscopic resection of the included studies was shorter than 15 years, which is considered the most favorable surveillance time for gastric cancer after LN dissection [35]. Finally, several analyses showed significant heterogeneity.

## Conclusions

In conclusion, after systematically reviewing and pooling the outcomes of additional gastrectomy, the present study showed that additional gastrectomy with LN dissection can prolong the 5-year OS, DFS and DSS of EGC after non-curative endoscopic resection. In addition, elderly patients can benefit from additional gastrectomy regarding 5-year OS. Therefore, additional gastrectomy with LN dissection might improve the survival of EGC after non-curative endoscopic resection. However, risk stratification should be performed to avoid excessive treatment. Re-ESD, even observation, might be the optimal choice for some populations.

## Authors’ contributions

R.C.N., S.Q.Y., Y.F.L., Z.W.Z. and Y.B.C. conceived of the study and participated in its design and coordination. R.C.N., S.Q.Y. and S.C. performed the statistical analyses and interpretation. R.C.N., Y.M.C., X.J.C. and G.M.C. drafted the manuscript. All authors read and approved the final manuscript.

## Funding

Not applicable.

## Supplementary Material

Supplementary Data
