# Supplementary material for: Additional gastrectomy in early-stage gastric cancer after non-curative endoscopic resection: a meta-analysis
Source: Gastroenterol Rep (Oxf). 2019 Mar 8;7(2):91–7. doi: 10.1093/gastro/goz007 (PMC6454843; doi:10.1093/gastro/goz007)
Supplement: Supplementary Data [file goz007_supplementary_data.doc]

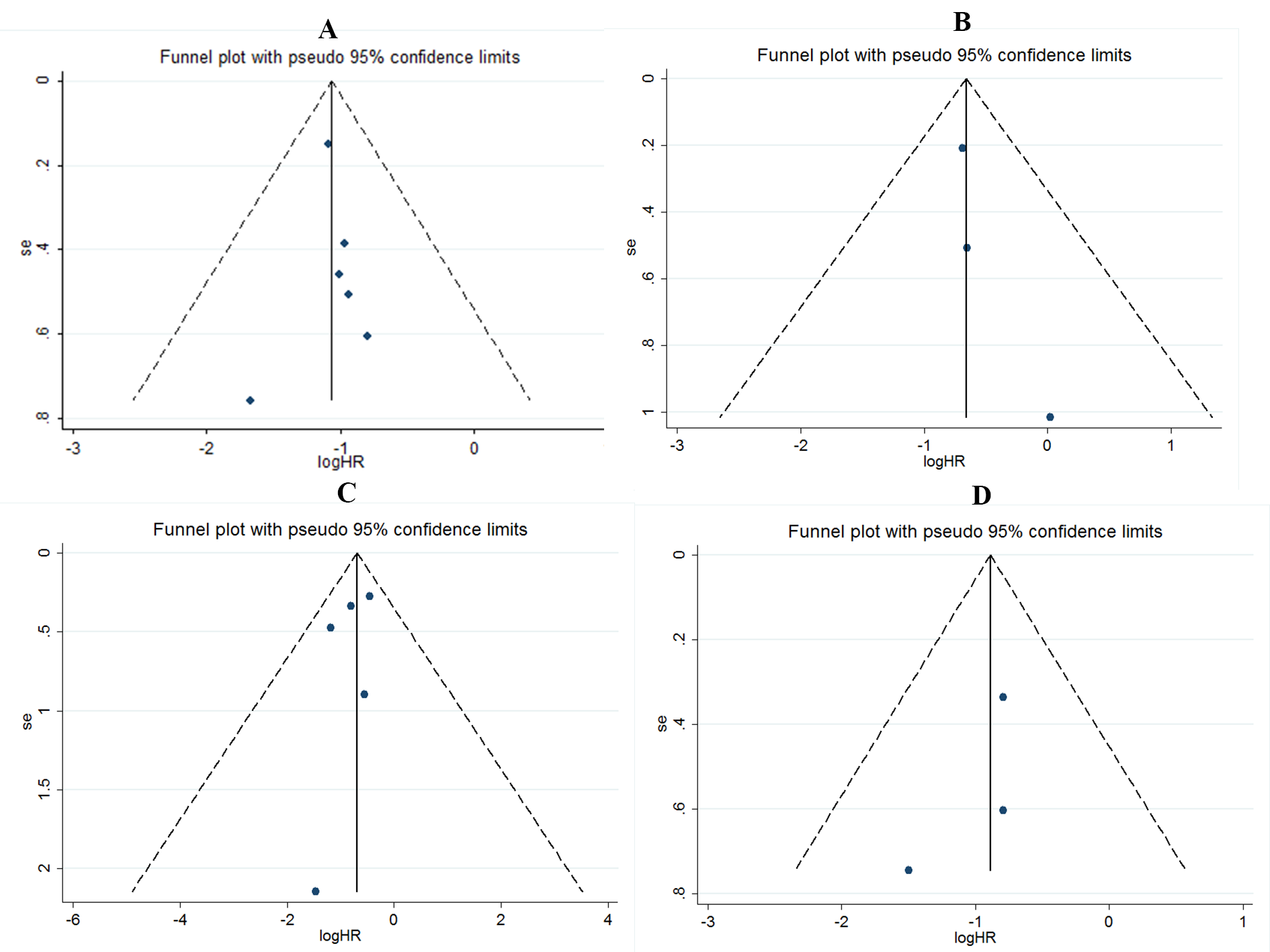


**Figure S1** Funnel plot for publication bias of overall survival (A), disease-free survival (B) and disease-special survival (C) meta-analysis in total analysis, and overall survival (D) in elderly patient subgroup.

**Table S1 Clinicopathological characteristics between the additional gastrectomy and non-gastrectomy groups.**

| Variables | No. of studies |  | Statistic | |  | Heterogeneity | |
| --- | --- | --- | --- | --- | --- | --- | --- |
|  |  |  | OR/WMD (95% CI) | P value |  | I^2^ | P value |
| Age (year) | 7[[9](#_ENREF_9), [14](#_ENREF_14), [18](#_ENREF_18), [22](#_ENREF_22), [24](#_ENREF_24), [25](#_ENREF_25), [31](#_ENREF_31)] |  | WMD: -4.51 (-6.80- -2.23) | < 0.001 |  | 89.5% | < 0.001 |
| Tumor location (lower third) | 5[[9](#_ENREF_9), [14](#_ENREF_14), [18](#_ENREF_18), [24](#_ENREF_24), [31](#_ENREF_31)] |  | OR: 1.02 (0.87-1.12) | 0.818 |  | 0 | 0.551 |
| Gross type (elevated type) | 5[[14](#_ENREF_14), [18](#_ENREF_18), [22](#_ENREF_22), [24](#_ENREF_24), [31](#_ENREF_31)] |  | OR: 0.99 (0.64-1.52) | 0.953 |  | 63.1% | 0.028 |
| Tumor size (cm) | 3[[9](#_ENREF_9), [18](#_ENREF_18), [24](#_ENREF_24)] |  | WMD: -0.24 (-0.57- 0.10) | 0.170 |  | 69.6% | 0.037 |
| Ulcer formation | 4[[14](#_ENREF_14), [18](#_ENREF_18), [24](#_ENREF_24), [31](#_ENREF_31)] |  | OR: 0.96 (0.50-1.83) | 0.889 |  | 70.5% | 0.017 |
| En bloc resection | 2[[9](#_ENREF_9), [31](#_ENREF_31)] |  | OR: 0.84 (0.45-1.57) | 0.592 |  | 0 | 0.372 |
| Tumor depth (SM1 stage) | 8[[9](#_ENREF_9), [14](#_ENREF_14), [16](#_ENREF_16), [18](#_ENREF_18), [22](#_ENREF_22), [24](#_ENREF_24), [25](#_ENREF_25), [31](#_ENREF_31)] |  | OR: 0.34 (0.23-0.50) | < 0.001 |  | 76.5% | < 0.001 |
| Undifferentiated type | 6[[14](#_ENREF_14), [16](#_ENREF_16), [18](#_ENREF_18), [22](#_ENREF_22), [24](#_ENREF_24), [31](#_ENREF_31)] |  | OR: 0.85 (0.71-1.02) | 0.078 |  | 46.2% | 0.098 |
| Lymphatic invasion | 3[[14](#_ENREF_14), [18](#_ENREF_18), [24](#_ENREF_24)] |  | OR: 2.54 (1.56-4.16) | < 0.001 |  | 64.7% | 0.059 |
| Horizontal margin | 3[[9](#_ENREF_9), [14](#_ENREF_14), [31](#_ENREF_31)] |  | OR: 0.83 (0.34-2.06) | 0.695 |  | 83.2% | 0.003 |
| Vertical margin | 5[[9](#_ENREF_9), [14](#_ENREF_14), [16](#_ENREF_16), [18](#_ENREF_18), [31](#_ENREF_31)] |  | OR: 2.20 (0.98-4.93) | 0.055 |  | 93.1% | < 0.001 |

OR, odds ratio; WMD, weighted mean difference; CI, confidence intervals; SM1 stage, tumor invasion < 500 µm into the submucosa;
